# Biological Nanoparticles for Enhancing Chronic Wound Regeneration

**DOI:** 10.3390/cells14201637

**Published:** 2025-10-21

**Authors:** Daniil Zotikov, Natalia Ponomareva, Sergey Brezgin, Anastasiia Kostyusheva, Anastasiya Frolova, Vladimir Chulanov, Alexander Lukashev, Peter Timashev, Dmitry Kostyushev

**Affiliations:** 1 Laboratory of Genetic Technologies, Martsinovsky Institute of Medical Parasitology, Tropical and Vector-Borne Diseases, Sechenov University, Moscow 119435, Russia; daniilzotikov@mail.ru (D.Z.); seegez@mail.ru (S.B.); kostyusheva_ap@mail.ru (A.K.); 2 Laboratory of Experimental Therapy of Infectious Diseases, Martsinovsky Institute of Medical Parasitology, Tropical and Vector-Borne Diseases, Sechenov University, Moscow 119435, Russia; ponomareva.n.i13@yandex.ru (N.P.); frolanasta@gmail.com (A.F.); vladimir@chulanov.ru (V.C.); 3 Engelhardt Institute of Molecular Biology, Russian Academy of Science, Moscow 119991, Russia; 4 Department of Infectious Diseases, Sechenov University, Moscow 119435, Russia; 5 Martsinovsky Institute of Medical Parasitology, Tropical and Vector-Borne Diseases, Sechenov University, Moscow 119991, Russia; alexander_lukashev@hotmail.com; 6 Research Institute for Systems Biology and Medicine, Moscow 117246, Russia; 7 Institute for Regenerative Medicine, I. M. Sechenov First Moscow State Medical University, Moscow 119991, Russia; timashev_p_s@staff.sechenov.ru; 8 Faculty of Bioengineering and Bioinformatics, Lomonosov Moscow State University, Moscow 119192, Russia

**Keywords:** regeneration, infection, anti-bacterial, VEGF, bioprinting, scaffolds, diabetic ulcer, immunity

## Abstract

**Highlights:**

**What are the main findings?**

- Biological nanoparticles (BNPs), demonstrate efficacy in chronic wound models by promoting immunomodulation, angiogenesis, and cell proliferation, while enabling targeted drug delivery.
- Advanced delivery platforms significantly enhance the stability, sustained release, and functional activity of BNPs in the challenging wound microenvironment, overcoming key limitations of current wound therapy.

**What is the implication of the main finding?**

- BNPs represent a therapeutic strategy with the potential to overcome the limitations of conventional chronic wound treatments, offering a biocompatible and multifunctional approach for managing complex, non-healing wounds.
- The ongoing clinical trials and development of engineered BNPs pave the way for standardized, scalable production, bringing nanotherapies closer to clinical reality and revolutionizing personalized wound care.

**Abstract:**

Chronic wounds (CWs) represent a growing global health concern with profound clinical and socioeconomic implications. Studies indicate that approximately 15% of CWs remain unhealed one year after the initial treatment. At the same time, it is assumed that from 1% to 2% of the population of developed countries will suffer from chronic wounds during their lifetime. CWs severely impair patients’ quality of life. Current therapies (compression bandages, antibiotics, hyperbaric oxygen, and skin grafts) face limitations, including toxicity, contraindications, inefficacy in patients with comorbidities like diabetes, and high cost. Biological nanoparticles (BNPs), particularly extracellular vesicles (EVs), emerge as transformative solutions due to their innate biocompatibility, targeted biodistribution, and multifunctional regenerative properties. This review examines the mechanisms by which BNPs promote CW healing and drug delivery. Innovative BNP delivery platforms (chitosan hydrogels, alginate films) are evaluated, enabling sustained release and responsiveness to the wound microenvironment. Clinical advances, including exosome-laden hydrogels that accelerate healing in diabetic ulcers, underscore BNPs’ potential to overcome conventional therapy limitations. By addressing the challenges of both pathophysiological complexity and healthcare system burden, BNPs demonstrate the potential to improve patient outcomes in the management of chronic wounds.

## 1. Introduction

**Figure 1:**
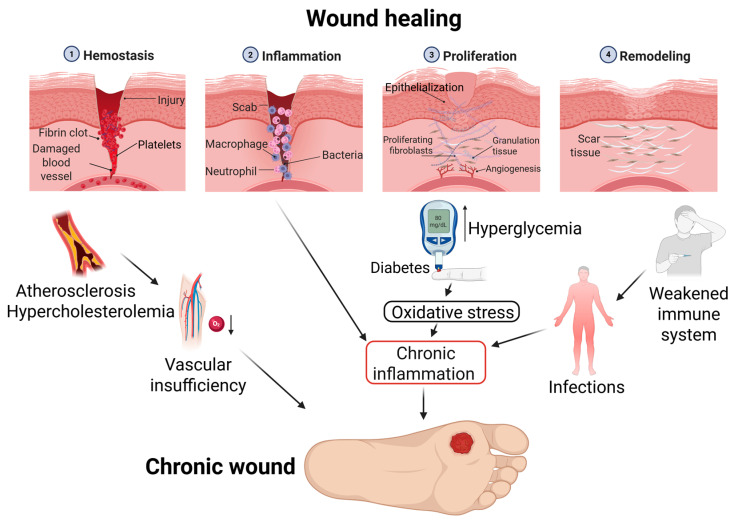
Multifactorial pathophysiology underlying chronic wound development. The core mechanism involves a self-perpetuating inflammatory feedback loop, preventing progression beyond the inflammatory stage of healing. This dysregulated state is driven by concurrent pathological processes: tissue ischemia due to macro- and micro-vascular insufficiency, hyperglycemia-induced endothelial dysfunction, and impaired immunity. These interconnected factors collectively inhibit cellular proliferation and tissue remodeling, resulting in a treatment-refractory chronic wound.

CWs are tissue injuries that do not progress through the standard stages of healing (hemostasis, inflammation, proliferation, remodeling) for 4–12 weeks despite treatment (Figure 1) [1]. They become stalled in the inflammatory phase, which prevents recovery. There are different causes and risk factors for CWs [2,3]. First of all, vascular insufficiency manifesting against the background of atherosclerosis and hypercholesterolemia is a powerful predictor of negative outcomes, because poor blood circulation prevents tissues from getting enough nutrients [4,5] and oxygen. High sugar levels in people with diabetes also damage blood vessels, which affects the tissue supply system [6]. Hyperglycemia inhibits the activity of antimicrobial peptides, such as cathelicidin [7], disrupts the barrier function of the skin, and promotes the formation of biofilms and pressure injuries [8]. In patients with limited mobility, the risk of adverse outcomes increases. A weakened immune system also increases the risk of delayed healing or wound infection. Infections and antibiotic resistance contribute to the formation of CW [9]. Additionally, the alkaline environment of wounds (for example, in diabetes) retards healing [10].

Local hypoxia and the alkaline pH of the wound environment are also key factors impairing healing. Oxygen is essential for the bactericidal activity of neutrophils, angiogenesis, and collagen synthesis, and its deficiency supports chronic inflammation. Biological nanoparticles (BNPs) offer a strategy for correcting these disorders. Exosomes, for example, are capable of delivering angiogenic factors (such as vascular endothelial growth factor, VEGF) to improve oxygenation, as well as antioxidants or pH-buffering systems. This allows BNPs to purposefully modulate the wound microenvironment, facilitating the transition from the stage of inflammation to regeneration [11].

Chronic wounds significantly reduce the quality of life of patients. Damage to blood vessels and nerves can cause chronic pain or, as in the case of diabetes, no pain at all, which is even more dangerous. If left untreated, CWs lead to systemic infections (meningitis, endocarditis) and other complications [12,13].

Over the years, many methods of CW treatment have been developed, but all of them have different limitations. Compression therapy (stockings/bandages), while effective for venous leg ulcers by counteracting venous hypertension, is contraindicated in patients with arterial insufficiency due to the risk of ischemic injury. Topical and systemic antibiotics face efficacy limitations due to biofilm persistence and microbial resistance. Prolonged systemic antibiotic use risks dysbiosis, organ toxicity (renal/hepatic), and allergic reactions, while indiscriminate topical application may cause contact dermatitis without improving healing rates [14,15]. Debridement (surgical, enzymatic, or biosurgical) is essential for removing necrotic tissue and biofilms but often requires anesthesia due to pain and may result in bleeding [16].

Advanced treatment modalities also have constraints. Hyperbaric oxygen therapy promotes oxygenation but is cost-prohibitive and equipment-dependent, and the evidence for efficacy is largely confined to ischemic diabetic ulcers, with limited long-term benefit. Negative pressure wound therapy (NPWT) enhances granulation but limits patient mobility, causes noise discomfort, and dressing changes may provoke pain/bleeding. Skin substitutes and grafts accelerate closure in refractory wounds but require viable wound beds with adequate perfusion; high cost and variable incorporation in ischemic tissue further restrict their use [14].

Critically, most interventions depend on addressing underlying pathologies (revascularization for ischemia, offloading for diabetic ulcers). Failure to correct these fundamental drivers of non-healing renders even advanced therapies ineffective, highlighting the need for personalized, multifaceted care [17]. Therefore, medicine is in need of innovative solutions, such as the use of pro-healing molecules delivered using nanotechnologies, including nanoparticles (NPs) of different natures. Different types of NPs are being studied in CWs treatment, for example: metal NPs (silver [18,19], copper [20], gold [21], and zinc oxide [22,23,24] NPs), polymeric NPs (chitosan [25,26,27], PLGA [28,29], and other), carbon-based NPs (graphene oxide [30] and carbon nanotubes [31]), lipid NPs (liposomes [32,33], micelles [34]), biological NPs (extracellular vesicles [35,36,37,38], including plant-derived exosome-like nanovesicles (PELNs) [39], extracellular vesicle-mimetic nanovesicles (EMNVs) [40,41,42,43], biomimetic nanoparticles [44], etc.). Non-biological particles were effective in experimental research; there are also active developments for them to create compatible delivery systems, such as patches [45], but most of them have significant disadvantages. Metal nanoparticle ions are toxic and can accumulate in organs. Metal oxides are insoluble, which leads to long-term persistence and ecotoxicity. Polymer nanoparticles have the risk of an immune response (as shown for PEG NPs), as well as difficulties in controlling the release [46]. Compared to other particles, BNPs have many advantages, such as high biocompatibility and biodegradability, packaging capacity, safety, ability to cross biological barriers, and programming via genetic or chemical methods [47].

The main types of BNPs used to treat CWs are extracellular vesicles (EVs), the most common of which are exosomes. Exosomes are naturally secreted, nanosized particles (up to 150 nm) that form via a complex mechanism involving multivesicular bodies [48,49]. The primary sources of exosomes for CWs treatment are mesenchymal stem cells (MSCs) [38,50], fibroblasts [51], keratinocytes [52], and immune cells [53]. Their composition is naturally suited to promote regeneration: MSC-derived exosomes are enriched with angiogenic factors (VEGF) and immunoregulatory microRNAs (miR-21) [54]. In contrast, plant EVs isolated from fruits, vegetables, or cereals form primarily through direct budding from the plasma membrane (similar to mammalian ectosomes) and differ fundamentally in composition: they are rich in phytosterols (e.g., sitosterol), secondary metabolism enzymes (enzymes, which are essential but not vital for the survival of the organism, such as cyclases, cytochrome P450 monooxygenases, etc.), and interspecies regulatory miRNAs, which can suppress pathogen genes [55]. To overcome the limitations of natural vesicles (e.g., heterogeneity and purification complexity [47]), engineered vesicle-mimetic nanovesicles (EMNVs) are being developed. These artificial nanovesicles are produced by cell fragmentation and subsequent reassembly into new particles, encapsulating surrounding molecules via extrusion, cavitation, microfluidics, and other methods [47]. EMNVs exhibit biological and chemical characteristics similar to those of exosomes [56], while offering standardized size, controlled composition, and enhanced scalability for therapeutic applications [57].

While the aforementioned non-biological NPs have demonstrated efficacy in pre-clinical settings, their clinical translation for chronic wound management is limited. The primary concerns for synthetic NPs include long-term toxicity, bio-persistence, unpredictable immune reactions, and long-term safety. Furthermore, the inherent heterogeneity of both NPs and chronic wounds themselves complicates the establishment of standardized potency assays and dosing regimens. The high cost, limited scalability, and high-quality production also present significant barriers to widespread clinical adoption. In contrast, biocompatibility and low immunogenicity are inherent advantages of BNPs. However, this does not make them superior in all aspects; natural EVs face challenges in scalable production, purification processes, as well as storage. Engineered alternatives like EMNVs and biomimetic NPs are emerging to solve these problems, offering controlled manufacturing at the potential cost of reduced biological complexity. For this to happen, careful particle safety studies must be conducted. Their inherent biological activity could potentially provoke unintended immune responses due to insufficient purification and the carry-over of mitochondrial or genomic DNA. Therefore, continued technological advancement is expected to yield more advanced BNPs capable of targeted drug delivery, high reproducibility, and minimal adverse effects.

In this review, we will look at the main mechanisms by which BNPs promote the healing of chronic wounds. The focus will be on their immunomodulatory properties and ability to stimulate regenerative processes (including angiogenesis, cell proliferation, and matrix remodeling), as well as their role in the delivery of therapeutic agents. In addition, innovative approaches to targeted delivery of BNPs to the wound area, modern research, and clinical trials will be discussed.

## 2. Mechanisms of BNP Effect on Wound Healing (Figure 2)

### 2.1. Immunomodulation

Exosomes stimulate the differentiation of Th1 and Th2 cells through their associated cytokines (IL-12 for Th1, IL-4 for Th2). By reinforcing T-cell regulatory pathways, they promote immune balance and reduce excessive innate immune activation and inflammation. Moreover, the introduction of exosomes to mice significantly reduced serum levels of key pro-inflammatory (TNF-α) and anti-inflammatory (IL-10) cytokines [58]. This could be provided by the ability of MSCs to switch macrophages from the pro-inflammatory phenotype M1 (producing TNF-α, IL-6) to the anti-inflammatory phenotype M2 (producing IL-10 and TGF-β). This occurs due to the transfer of signaling molecules (lipids, miRNAs, proteins) from BNPs to macrophages [59,60]. In particular, EVs of royal jelly (RJ-EVs) reduce the production of pro-inflammatory cytokines (TNF-α, IL-6) and enhance the secretion of anti-inflammatory cytokines (IL-10, TGF-β1) in macrophages, which was associated with the presence of MRJP1-3 proteins and fatty acids (10-HDA, 10-HDAA, SEA), known for their anti-inflammatory properties [61].

**Figure 2:**
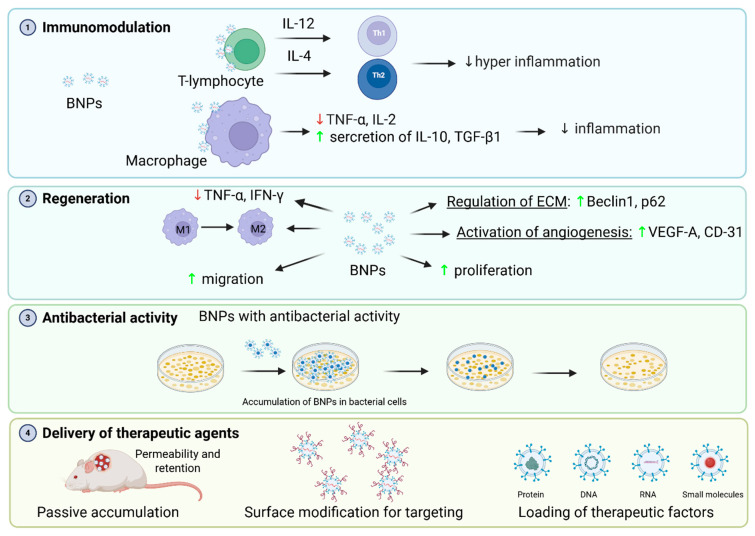
Multimodal mechanisms of BNPs in wound healing. BNPs, encompassing natural exosomes and engineered nanocarriers, facilitate the repair of chronic wounds by concurrently targeting multiple pathological barriers through synergistic mechanisms. Immunomodulation: BNPs resolve persistent inflammation by promoting macrophage polarization from a pro-inflammatory (M1; TNF-α, IL-6) to an anti-inflammatory (M2; IL-10, TGF-β) phenotype. They further restore immune equilibrium by modulating T-helper cell differentiation and balancing Th1/Th2 responses. Regeneration: BNPs activate pro-angiogenic signaling pathways, upregulating vascular markers VEGF and CD3. They concurrently enhance the proliferation and migration of fibroblasts and endothelial cells. Additionally, they orchestrate ECM remodeling by regulating matrix metalloproteinase activity (e.g., MMP-9). Antibacterial Activity: Engineered BNPs can directly accumulate at infection sites and induce antibacterial activity, disrupting bacterial cells. Targeted Therapeutic Delivery: BNPs passively accumulate in wounds via the enhanced permeability. Active targeting is achieved through surface functionalization with ligands for specific receptor binding. This enables spatiotemporally controlled delivery of diverse cargo, including nucleic acids (DNA, miRNA), proteins (e.g., growth factors), and small-molecule drugs to the injury site.

### 2.2. Regeneration

BNPs accelerate wound healing through diverse effects:

(1) Modulation of the inflammatory response by suppressing pro-inflammatory cytokines: M2 macrophage exosomes (MEs) and exosome-like nanoparticles (ELNs) from a cockroach (*Periplaneta americana)* (PA-ELNs) reduce TNF-α, IL-6, and IFN-γ levels, thereby mitigating chronic inflammation in diabetic wounds. MEs and PA-ELNs also promote macrophage polarization by upregulating CD206 and arginase-1 (Arg1) expression [62,63].
(2) Stimulation of angiogenesis by increased expression of angiogenic factors: MEs activate the Wnt/β-catenin pathway, increasing VEGF-A and CD31 levels to promote the formation of new blood vessels [62]. PA-ELNs regulate the signaling pathways of TGF-β and mTOR, enhancing VEGF and CD31 expression [63]. MSCs stimulate angiogenesis by activating the AKT/eNOS signaling pathway through an increase in the level of miR221-3p, which enhances the proliferation, migration, and tubule formation of endothelial cells, as well as the secretion of angiogenic factors (VEGF, PDGF, bFGF, ANG1) [64]. Studying adipose-tissue-derived stem cells (ASCs) also showed positive results on the relationship of PDGF and bFGF signaling pathways and angiogenesis [65].
(3) Acceleration of cell proliferation and migration: PA-ELNs increase the proliferation of fibroblasts (L929) and endothelial cells (HUVECs) by 50–60% [63]. Exosomes derived from 4T1 mouse breast carcinoma cells (TEXs), as well as ginseng-derived nanoparticles (GDNPs), also showed positive effects. TEXs enhance the proliferation of fibroblasts and endothelial cells through activation of the PI3K/Akt pathway, and GDNPs increase the proliferation of keratinocytes, fibroblasts, and endothelial cells by 20–40%, and accelerate the S-phase of the cell cycle [66,67].
(4) Extracellular Matrix (ECM) remodeling: BNPs help rebuild the structural scaffold of the skin. They promote healthy tissue turnover by regulating processes like autophagy and, crucially, by inhibiting enzymes that would otherwise break down the newly formed matrix. This ensures the rapid and strong development of granulation tissue [63].
(5) Delivery of bioactive molecules: exosomes carry microRNAs (for example, miR-21, miR-146a) that suppress inflammation and activate regenerative pathways, and PA-ELNs contain proteins involved in ion transport and cell adhesion [62,63].

### 2.3. Antibacterial Activity

BNPs can be combined with special therapies to provide antibacterial activity. For instance, M2/IR780@PLGA NPs feature a core made of the biocompatible polymer PLGA, which encapsulates IR780 sonosensitizers. When activated by ultrasound, these sonosensitizers generate cytotoxic reactive oxygen species (ROS). The ROS destroy bacterial membranes and inhibit biofilm formation. A key feature of these NPs is their shell, derived from M2 macrophage membranes. This shell enables selective accumulation at inflammatory sites via the CD206 receptor and improves tissue penetration. In vivo experiments have confirmed that M2/IR780@PLGA not only effectively destroys methicillin-resistant Staphylococcus aureus (MRSA) but also stimulates the expression of IL-10 and CD206, reducing CD86 levels and activating dendritic cells of the spleen. This provides a dual defense mechanism: direct ROS-mediated destruction of pathogens and immunomodulation that accelerates healing [68]. While M2/IR780@PLGA has a direct antibacterial mechanism, most BNPs can only indirectly provide antibacterial activity due to the hydrogels they are part of or due to their regenerative properties, creating an environment unfavorable for infection. For example, chitosan-silk hydrogel retains moisture, provides gradual release of exosomes, and provides mechanical protection of the wound [69].

### 2.4. Delivery of Therapeutic Agents

Exosomes and their hybrids with liposomes/nanoparticles are promising platforms for drug delivery to wounds. Genetic and chemical engineering strategies make it possible to create nanocarriers that specifically accumulate in the wound, delivering anti-inflammatory, antimicrobial, and regenerative agents [70]. BNPs (especially exosomes and their engineered counterparts) can be used to deliver therapeutic agents to the wound area in the following ways:

Nanoparticles of 30–150 nm in size are able to passively accumulate in areas of inflammation, including wounds, due to increased vascular permeability and impaired lymphatic drainage in damaged tissues (briefly termed increased permeability and retention) [71]. Exosomes of macrophages and neutrophils naturally migrate to the area of inflammation. Specific receptors that could be responsible for the migration process, such as CXCR2, were tested as migration factors, but the use of antibodies against CXCR2 did not affect the migration and tissue penetration processes, so the exact mechanism of targeting is not described in the articles [72,73]. In mouse studies, exosomes derived from the RAW 264.7 macrophage cell line accumulated in wounds and suppressed inflammation [74].

Biological and chemical modification methods for targeting also ensure accurate delivery of BNPs. Peptides specific to receptors in the wound environment (for example, integrin, growth factor receptors) may be genetically fused with exosome membrane proteins (LAMP-2B, CD63 [75], PTGFRN [76], and their genetic variants [43]). RGD-peptide (Arg-Gly-Asp) directs exosomes to αvβ3 integrins overexpressed in the endothelium of wound vessels, stimulating regeneration [74]. Chemical modification allows covalently binding ligands to the surface of exosomes through lipid anchors (DSPE-PEG, cholesterol). DSPE-PEG-RGD increased the accumulation of exosomes in tumors, which is applicable for wounds with overexpression of integrins [77].

In addition to drugs, different types of biologicals can be loaded into exosomes [78,79]. Encapsulation in exosomes protects proteins from degradation; thus, it is possible to deliver various growth factors: VEGF, TGF-β, and catalase. Catalase-loaded exosomes reduced oxidative stress in models of neurodegeneration, which is relevant for chronic wounds.

Combining the benefits of exosomes and liposomes improves loading and delivery efficiency. Hybrids of exosomes combined with liposomes deliver CRISPR/Cas9 in MSCs for gene editing, RNA molecules for gene knockdown, or, potentially, mRNA delivery [43,80,81], which can be used to regulate inflammation in wounds [77,82].

## 3. Innovative Technologies for Delivering NPs to the Wound Area

Hydrogels are three-dimensional polymer networks capable of retaining large amounts of water (up to 99% by weight). They combine the properties of solid materials (mechanical stability) and liquids (transport of molecules). In biomedicine, they are used as scaffolds for the delivery of therapeutic agents (cells, proteins, exosomes) due to their biocompatibility, adaptivity to tissues, and usability for controlled release. Hydrogels serve as carriers for biological nanoparticles [83] in the treatment of wounds and have useful properties: the gel is formed in situ directly in the wound, filling uneven contours, while the pores of the hydrogel regulate the diffusion of particles. For example, exosomes were released from chitosan hydrogel for 14 days [84], and the hydrogel network prevented degradation of nanoparticles in an aggressive wound environment (for example, in diabetic inflammation). Hydrogels have their advantages, such as improved integration with tissue, because the predominance of water in the composition imitates the ECM [85]. In addition, the slow release of exosomes/growth factors accelerates angiogenesis and re-epithelialization over time, providing a prolonged effect [84]. Hydrogels may include additional components: chitosan [84] and other antimicrobial agents, or signaling molecules such as VEGF [85]. They also have their disadvantages: insufficient data on the long-term effects on organs (liver, kidneys) after hydrogel degradation in vivo; the difficulties of standardization and quality control due to the instability of hydrogels; gelatin hydrogels liquefy at temperatures > 35 °C, and control over the release of nanoparticles is not always straightforward. The released nanoparticles are quickly washed out of the wound by exudate, especially without a carrier. In case of non-healing wounds, hydrogels may have limited effectiveness; in the conditions of hyperglycemia, infections, and impaired angiogenesis, their efficacy may be reduced. Moreover, the process of exosome isolation (ultracentrifugation) and hydrogel synthesis is laborious, and the shelf-life of hydrogels is limited, which also provides technological and production problems [37,86].

Wound treatment films are thin polymer matrices designed for local delivery of therapeutic agents (including nanoparticles) into the wound environment. BNPs can be encapsulated in the matrix during film formation and then released in a controlled manner due to diffusion or degradation of the polymer. They also have various advantages: the films demonstrate low immunogenicity and lack of irritation, serve as a physical barrier against infections, and prevent tissue adhesion, which protects the wound surface. The disadvantages of films loaded with BNP include limited nanoparticle loading capacity, poor retention of hydrophobic agents in hydrophilic matrices, difficult multi-stage manufacturing, and a need for frequent film replacement due to the lack of pores and limited gas exchange [29,87,88]. Compared to hydrogels, films adapt less well to the wound due to stiffness, degrade more slowly, and have a risk of immune rejection, while hydrogel’s ECM mimics the native matrix. A critical, yet under-investigated, aspect is the long-term fate of these delivery systems in vivo. The degradation products of hydrogels and polymer films, while often designed to be biocompatible, can accumulate in organs such as the liver and kidneys. Potential immune reactions or chronic inflammation at the sites of accumulation of decay products are key points for further clinical research.

Another potential BNP carrier is alginates, natural polysaccharides obtained from brown algae and bacteria. Alginates can be processed into various forms such as hydrogels, microspheres, fibers, and sponges, making it possible to transport drugs and biological nanoparticles. Highly purified forms of alginates do not cause a significant immune response, which is critical for long-term use. They are able to absorb 15–20 times more exudate than their own weight, maintaining an optimally moist wound environment, which accelerates tissue regeneration and facilitates the controlled release of BNPs [89,90]. In some cases, alginate hydrogels can exhibit their own healing properties via macrophage activation and promotion of IL-6 and TNF-α production [91]. However, pure alginate hydrogels are brittle and easily break down under stress, while the hydrophilicity of alginate promotes the “washing out” of BNPs in the first hours after application [90]. Moreover, alginate dressings lose their structural integrity upon contact with Na^+^ ions in wound exudate, which can lead to premature release of BNPs [89].

BNPs can also be delivered using microneedles (MN), miniature needles (100–1000 microns long) integrated into patches or hydrogel matrices. In the context of wound treatment, they painlessly penetrate the epidermis/dermis, delivering BNPs to the deep layers of the wound and providing a load of biological agents mimicking the effects of injected whole-cell MSCs. The advantages of this delivery method include the lack of pain (needles do not reach nerve endings) and a reduced risk of secondary damage. Also, the unique structure allows separating functions: the tips of the needles release exosomes into the deep layers of the wound, while the base can be loaded with other particles. The main disadvantages of this method are the need to achieve optimal density and mechanical strength, which requires precise biodesign, and low agent loading, which requires frequent replacement of patches [92,93]. The main types of advanced delivery platforms, along with their key characteristics, are summarized in Table 1.

## 4. Models for CW Research

**Figure 3:**
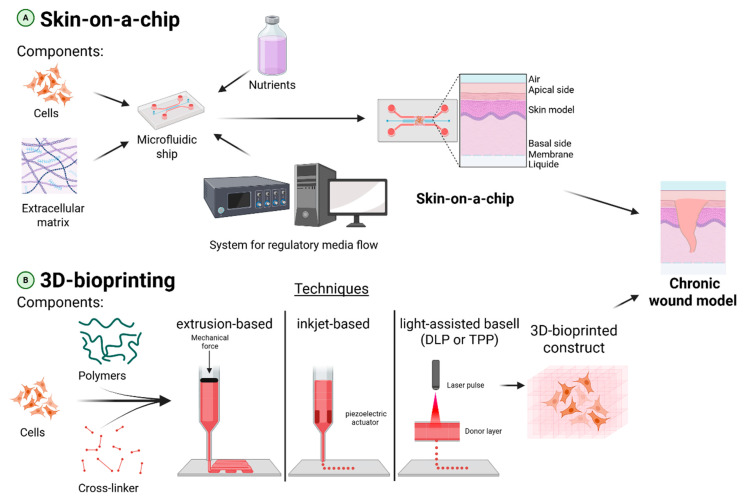
Advanced engineered models for chronic wound research. (**A**) Skin-on-a-Chip: This microphysiological system integrates human-derived cells, ECM components, and a perfused circulatory system within a microfluidic chip. It accurately mimics key aspects of skin physiology and the wound microenvironment. The dynamic flow of nutrients and biochemical factors allows for real-time, high-resolution analysis of cell-cell interactions, host-microbiome crosstalk, and drug responses under physiologically relevant conditions. (**B**) 3D Bioprinting: These techniques enable the construction of sophisticated, multi-cellular wound models with high architectural precision. Extrusion-based bioprinting employs mechanical pressure to continuously deposit bioink filaments, making it ideal for creating large, structurally robust constructs. Inkjet-based bioprinting utilizes thermal or acoustic forces to eject picoliter droplets of bioink, enabling high-speed printing for rapid prototyping. Light-assisted bioprinting (e.g., DLP/TPP) uses projected light patterns to photopolymerize photosensitive bioinks in a layer-by-layer fashion, achieving the highest resolution for complex microarchitectures. Collectively, these technologies enable the production of more realistic models of wound healing.

There are many different models that have specific applications for studying certain processes or stages of wound healing (Figure 3). In silico models offer the possibility of modeling spatiotemporal interactions and concentration gradients without expensive animal experiments, but they seriously simplify reality by ignoring the three-dimensional architecture of tissue and wound depth and are often limited only to modeling the innate immune response [95,96]. The simplest 2D in vitro models, despite their low cost and speed, have low reproducibility, do not take into account intercellular interactions and the three-dimensionality of the in vivo microenvironment, and are difficult to use for studying chronic processes [97,98]. Standard studies, such as the CCK-8 assay, are usually performed on 2D models to assess cell viability and proliferation, and the Tube formation assay is used to evaluate the ability of endothelial cells to form tubular structures. Popular tests such as the scratch assay and the trans-well migration assay are used to assess the ability of cells to migrate. More advanced 3D models (spheroids) make it possible to recreate cell-matrix interactions and the metabolic gradient, including hypoxia in the nucleus, which makes them promising for regenerative medicine, but they require high labor costs and carry the risk of necrosis in the center of large spheroids [99,100,101]. In vivo models demonstrate the greatest physiological relevance, as they allow studying the healing process in an organism with a functioning immune system and systemic factors. For example, the diabetic animal model is suitable for screening therapeutic interventions in conditions simulating metabolic disorders [102,103,104], while the ischemic model is ideal for isolated studies of the effect of hypoxia on impaired tissue repair [105,106]. A wound model with a recreated biofilm is important for assessing the dynamics of a persistent infection and for real-time antibiotic testing, although it is highly expensive [107,108]. Thus, no model is ideal, and the choice should be determined by a specific scientific question. Complex in vivo models (ischemic, diabetic, infected) are preferred for studying systemic effects and complex pathophysiological mechanisms, while in silico or advanced 3D models can be used at the initial research phase for high-throughput screening or investigation of mechanisms.

Currently, a promising area involves developing more complex, human-oriented systems. These include skin-on-a-chip (microfluidic devices that mimic tissue physiology) [109], which have since evolved into more advanced organ-on-a-chip models [110,111]. Another key technology is 3D bioprinting [112,113], which allows for high-precision control over cellular composition and architecture. By integrating multiple cell types, such as fibroblasts, immune cells, and even the microbiome, these technologies aim to create the most complete in vitro models of chronic wounds. At the moment, there are many new techniques that allow 3D bioprinting, among them are inkjet-based bioprinting [114], extrusion-based bioprinting [115,116], light-assisted bioprinting, which uses light for layer-by-layer curing of photosensitive liquid bio-inks [117], which in turn is divided into digital light processing (DLP)-based bioprinting [118] and two-photon polymerization (TPP)-based bioprinting [119]. Light-mediated methods are chosen when high detail is needed, while extrusion-based bioprinting is used to produce large and durable structures, and Inkjet-based bioprinting provides the highest speed of work. The development of new models and a detailed study of the advantages and disadvantages of existing models (Table 2) will allow a competent approach to choosing the appropriate model in the context of exploring the possibility of using BNPs for CW treatment.

## 5. Modern Research on BNPs in CW Treatment

Recent publications have demonstrated an increasing interest in the use of exosomes for CW treatment. The primary model of choice for studying the therapy of CWs is the diabetic wound model (Table 3). This choice is justified by the fact that diabetes is the leading cause of non-healing wounds and requires new approaches to combat the complications of the pathological process.

In one of the studies [126], a combined system based on exosomes encapsulating the adeno-associated virus encoding VEGF (EV-AAV from HEK293T) and exosomes of umbilical cord mesenchymal stem cells (hUC-MSC-Exo) integrated into the thermosensitive hydrogel FHCGgel was developed. In this study, application to diabetic animal wounds showed accelerated healing due to multiple factors: enhanced angiogenesis through endogenous VEGF expression, restoration of mitochondrial endothelial function, suppression of the cGAS-STING pathway (elimination of senescent macrophages), and an increase in Treg cells, which comprehensively remodels the inflammatory microenvironment.

An alternative source of exosomes is dedifferentiated fat cells (DFATs) [127] from human lipoaspirate, delivered in a GelMA hydrogel matrix. This strategy activates the Wnt/β-catenin pathway, stimulating fibroblast proliferation, endothelial cell migration, and angiogenesis, as well as a shift in the polarization of macrophages, which ensured rapid wound closure and improved collagen maturity in vivo. A different approach [128] focused on injectable native adipocyte exosomes (ADSCs): the key mechanism hypothetically involves the delivery of miR-204-5p, which targets TGF-β1 by inhibiting phosphorylation of Smad2/3 and suppressing fibroblast differentiation into myofibroblasts, leading to accelerated scar-free healing of diabetic wounds. Collectively, these studies [126,127,128] demonstrate the diversity of exosome sources, delivery forms, and molecular targets, highlighting their common potential to overcome the limitations of traditional chronic wound therapy through angiogenesis, immunomodulation, and fibrosis suppression.

Facilitating exosome production is another direction of research. Using 3D cell cultures instead of 2D cultures significantly slows down cell aging, thereby creating favorable conditions for sustainable exosome production [129]. In addition, 3D culture can enhance exosome secretion and enhance their angiogenic properties.

Storage, stability, and validation of activity are among the biggest challenges in the BNPs field [130,131,132]. The use of additional technologies to deliver therapeutic agents to the wound site ensures the protection of nanoparticles and the bioactive molecules contained within them. Despite the aforementioned disadvantages of using hydrogels, they remain the main technology for delivering BNPs in CW treatment (Table 3). An important indicator for delivery vehicles is the degradation period. Optimization of the hydrogel carrier biodegradation rate is aimed at creating a protective depot for BNPs, ensuring their stability and controlled delivery to the regeneration zone over the time required for the execution of cell-signaling cascades.

To solve the problem of rapid in vivo elimination of exosomes, an innovative hydrogel based on chitosan and polyethylene glycol (CS-PEG) and enriched with adipose tissue exosomes (ADMSCs) has been developed [133]. Furthermore, the use of the EV-AAV-VEGF hydrogel system in another research provided dual protection for both AAV and EVs [126]. Overall, hydrogels have been shown to provide controlled release of exosomes for up to 14 days, enhance endothelial cell migration, reduce oxidative stress, and stimulate angiogenesis, demonstrating good biocompatibility [126,127,133].

However, hydrogels also demonstrated limitations in experimental CW healing studies. A comparison of HaCaT-Evs (regular exosomes) with GelMA-EVs (exosomes in a hydrogel) [134] showed low statistical significance in all in vitro and in vivoexperiments (*p* < 0.05). This finding may be associated with the aforementioned disadvantages of hydrogels, but it also underscores a critical point for clinical translation: the added complexity of a delivery system must provide significant therapeutic benefit to justify manufacturing cost and potential risks associated with inflammatory responses. In some cases, as shown by the significant results from subcutaneous injections, the development of simpler, more direct delivery methods may offer a safer route to initial clinical adoption, even if they provide less controlled release.

At the same time, studies using subcutaneous injection of nanoparticles have shown statistically significant results (*p* < 0.001) in the treatment of chronic wounds in vivo [51,128,135]. These further highlight that exosomes are extremely effective in CW treatment even in the absence of special delivery systems.

While subcutaneous injections show efficacy in controlled animal studies, they may be less practical for widespread human use, especially for large or multiple wounds, due to patient discomfort and the need for professional intervention. Hydrogels offer a more user-friendly, topical application but must overcome their limitations of rapid washout and potential reduced efficacy in highly exuding wounds. The ideal delivery platform must not only protect and release BNPs in a sustained manner but also be easy to apply and acceptable to patients. The choice between injectable versus topical formulations will have significant implications for treatment protocols, patient compliance, and ultimately, commercial viability.

In addition to extracellular vesicles, EMNVs have potential in the treatment of chronic wounds [40]. It has been previously shown that this type of nanoparticle has a great capacity for loading and delivering biomolecules [43,136]. High loading efficiency and easier production optimization compared to exosomes make EMNVs more attractive for the treatment of diseases. While most exosome purification protocols rely solely on ultracentrifugation (Table 3), EMNVs in this study were purified using ultrafiltration and density gradient ultracentrifugation [40]. These approaches, combined with cell extrusion to obtain EMNVs, provide better size distribution, integrity (no aggregation and gravity-induced damage), and purity of the nanoparticles. Developing approaches to improve the production, purification, and stability of BNPs could expand their potential for use in CW treatment.

## 6. Clinical Trials

BNPs for the treatment of CWs are being actively investigated in clinical practice (Table 4). Pilot studies show promising results when using exosomes from various sources. For example, in one study [137], exosomes isolated from patients’ adipose tissue (200–300 mL in volume) were encapsulated in a hydrogel dressing. The use in 5 patients with wounds of different sizes (1–20 cm^2^) showed accelerated healing after 4 weeks [138]. An open study with 38 participants revealed that topical application of WJ-MSC-CM led to a decrease in ulcer size and increased granulation after just 2 weeks. Additionally, the effectiveness of plasma exosomes in difficult-to-heal ulcers (rheumatic, diabetic) was studied in 5 patients [139]. Monitoring for 28 days showed positive dynamics in reducing the size of wounds (length/depth) and reducing pain.

New combinations and methods of exosome delivery are also being tested. One of the current open randomized trials [140] is recruiting 20 patients with refractory wounds to compare combination therapy with adipose tissue exosomes (AT-EVs) with hyaluronic acid (HA) and HA monotherapy. The main checkpoint is the percentage of wound healing after 10 weeks. Another approach is being investigated in a study where 10 patients with long-term CW are injected with autologous exosomes (EVs) into the periulcerous zone [141]. In addition to standard therapy (sanitation, exofiber bandages, unloading), participants received 5 mL of EVs weekly, and the goal was to assess the healing rate. Results?

These studies, most of them ongoing, collectively highlight the diversity of exosome sources (adipose tissue, plasma, umbilical cord stem cells) and their application forms (dressings, gels, topical solutions, injections), demonstrating the potential of cell-free therapy in overcoming the limitations of traditional wound treatment methods.

## 7. Conclusions

CWs represent a significant and growing healthcare burden with a profound impact on patients’ quality of life. Current therapeutic strategies often fall short due to limitations such as toxicity, inefficacy in complex comorbidities, high cost, and systemic side effects. BNPs, particularly EVs, have emerged as a transformative therapeutic paradigm. Their innate biocompatibility, targeted biodistribution, and multifunctional regenerative properties, including potent immunomodulation, stimulation of angiogenesis and cell proliferation, ECM remodeling, and antimicrobial activity, directly address the complex pathophysiology of stalled healing. Innovative delivery platforms (e.g., hydrogels, films, microneedles) enhance BNPs’ efficacy by ensuring sustained release, protection within the wound environment, and responsiveness to local cues. Preliminary clinical data support the promise of BNP-based therapies in accelerating closure and improving outcomes in challenging wounds such as diabetic foot ulcers. While further large-scale clinical validation and refinement of scalable production are needed, BNPs hold immense potential to overcome the limitations of conventional treatments and revolutionize chronic wound management. Natural EVs are characterized by high heterogeneity, unpredictable cargo, and significant batch-to-batch variability in both composition and function. In contrast, BNPs offer superior homogeneity and controllability. While the safety profile of BNPs is inherently dependent on their final composition, and the incorporation of immunogenic components would increase their risk, rational design and advanced purification protocols can minimize contaminants like mitochondrial and genomic DNA. This is a distinct advantage, as even naturally produced EVs can harbor immunogenic DNA. Regarding long-term safety, no significant differences are anticipated between EVs and BNPs.

At the same time, BNPs are currently not in the market, and no FDA-approved BNPs-based drugs are available, mostly because the field is relatively new, but rapidly developing. The principal constraints for manufacturing BNPs are not technological, but rather commercial and biological. A high market entry barrier exists, as investors and manufacturers question the utility of a product that lacks a market precedent. Furthermore, obtaining sufficient cell culture mass presents obvious challenges, including the need for clean-room facilities, rigorous aseptic techniques, and high associated costs. Once the biomass is secured, however, downstream processing, including purification and BNP fabrication, leverages established, equipment-ready approaches.

A deeper understanding of the immunogenic risks associated with engineered BNPs and a comprehensive safety profile of the long-term in vivo degradation of advanced delivery platforms are critical next steps. Future research must prioritize these aspects to ensure that the next generation of BNP therapies is not only effective but also unequivocally safe for use in vulnerable patient populations. Significant research is currently underway to advance BNP development and refinement, and to establish robust methods for their standardization, analytical control, large-scale production, and storage, key steps toward enabling their market entry.

## Figures and Tables

**Table 1:** Advanced technologies for delivering NPs.

Type	Description	Advantages	Disadvantages	References
Hydrogels	3D polymer networks retaining high water content (up to 99%). Serve as scaffolds for NP delivery	Biocompatibility In situ formation adapts to wound contours. Sustained release of NPs. Pores regulate NP diffusion. Can incorporate antimicrobials (chitosan) or growth factors (VEGF).	Instability (gelatin hydrogels liquefy >35 °C). Rapid NP washout by exudate without carriers. Labor-intensive synthesis and short shelf life. Reduced efficacy in comorbidities (diabetes).	[37,83,84,85,86,94]
Films	Thin polymer matrices for local NP delivery. NPs are encapsulated during film formation, released via diffusion/degradation.	Low immunogenicity and no irritation. Physical tissue adhesion/barrier against infection. Controlled NP release.	Limited NP loading capacity. Poor retention of hydrophobic NPs in hydrophilic matrices. Poor adaptation to wound contours. Multi-stage manufacturing.	[29,87,88]
Alginates	Natural polysaccharides (from algae/bacteria) processed into hydrogels, films, or sponges for NP/drug delivery.	High exudate absorption (15–20× weight). Maintains a moist wound environment. Low immunogenicity (purified forms). Versatile forms (hydrogels, fibers).	Brittle structure (breaks under stress). Premature NP release due to Na+-induced degradation. Rapid NP washout (hydrophilicity).	[89,90]
Microneedles	Miniature needles (100–1000 μm) on patches/hydrogels. Penetrate epidermis/dermis to deliver NPs (e.g., exosomes, MSCs) to deep layers.	Minimally invasive and painless (avoids nerve endings). Dual-function design: tips (exosomes) + base (other NPs). Reduces secondary damage.	Low NP loading capacity (frequent replacement needed). Complex biodesign for optimal strength/density. Manufacturing precision required.	[92,93]

**Table 2:** Types of models.

	Model Name	Characterizes	Advantages	Disadvantages	Ref.
In silico	Agent-Based Model based on Glazier–Graner–Hogeweg method, also known as Cellular Potts Model	The model is a spatiotemporal simulation of the immune response after a burn	(1) Allows modeling of localized interactions and concentration gradients (2) New cell types, cytokines, and interaction rules can be added (3) Parameters are tuned based on real biological data	(1) Requires significant resources with a large number of agents (2) Many processes (e.g., apoptosis, proliferation) are specified probabilistically or omitted (3) Does not take into account the three-dimensional structure of the tissue and the depth of the wound (4) The model focuses only on the innate immune response	[95,96]
	Molecular docking and ADME prediction	Using AutoDock 4.2, pkCMS, SwissTargetPrediction software, AutoDock, iGemdock, and modeling	(1) Fast and low cost (2) Prediction of mechanisms of action at the molecular level (3) Ability to screen thousands of compounds virtually	(1) Does not replace experimental data (2) Does not take into account complex biochemical contexts in vivo	[120,121]
In vitro	2D cell model	A method in which a cell monolayer is mechanically damaged, after which the rate of healing of the resulting lesion, simulating a wound, is measured	(1) Ease of implementation and availability of equipment (2)Low cost and high speed of experiments	(1) Low reproducibility due to the subjectivity of damage application (2) Short-term nature of the model (3) Does not take into account the three-dimensional architecture and intercellular interactions typical of in vivo conditions Does not involve immune mechanisms?	[97,98]
	3D cell culture (spheroids)	Mesenchymal stromal/stem cells from various sources cultured under low adhesion conditions, resulting in self-assembly of cells into multicellular aggregates—spheroids.	(1) Reproduces cell-cell and cell-matrix interactions. (2) Suitable for studying in vivo-like processes: modeling hypoxia and metabolic changes in the center of the spheroid. (3) Can be used in regenerative medicine and for wound healing	(1) Labor-intensive and expensive compared to 2D culture. (2) Cells in the center may already undergo cell death due to ischemia and lack of nutrients. (3) Risk of necrosis in the center of large spheroids due to a lack of oxygen	[100,101]
In vivo	Diabetic wound model	The model involves artificial induction of diabetes in various ways, like a combination of a high-fat diet and low doses of streptozotocin	(1) Possibility of studying long-term wound healing in diabetic conditions. (2) Suitable for screening therapeutic interventions	(1) STZ toxicity may affect animal health and survival. (2) Limited translation to humans due to species differences in wound healing and metabolism.	[102,103,104]
	Ischemic wound model	Ischemic wound models allow studying the mechanisms of impaired healing under conditions of hypoxia, oxidative stress, and age-related changes	(1) It is possible to study ischemic and non-ischemic wounds simultaneously on one animal (2) The model allows for isolating the effect of hypoxia from other factors.	(1) The model requires surgical intervention, which may affect the general condition of the animal (2) Despite physiological similarities, the response to ischemia in humans may differ	[105,106]
	Biofilm-infected wound model	The model represents a long-term polymicrobial biofilm infection of full-thickness burn wounds in different animals	(1) Takes into account the immune response and systemic factors (2) The ability to assess the dynamics of infection and treatment in real time.	(1) High cost and labor intensity. (2) Possible variability between animals.	[107,108]
	Pressure wound model	Model reproduces the key mechanism of pressure ulcer formation in humans—repeated cycles of ischemia and subsequent reperfusion	(1) The most accurate simulation of the mechanism of pressure ulcer formation in humans (2) Does not require surgical intervention (3) Allows tracking all stages of the process—from injury to complete healing	(1) The model creates an acute injury, while clinical pressure ulcers are often the result of long-term chronic pressure. (2) The model was tested on young and healthy animals, while in humans, pressure ulcers are more likely to occur due to old age, diabetes, circulatory problems, and immobility	[122,123]
	Ulcer wound model	A model based on subcutaneous iron administration to induce hemosiderin deposits, oxidative stress, and delayed wound healing mimicking human chronic venous insufficiency	(1) Better reproduces the pathology of chronic ulcers in humans due to abundant exudation, inflammation, and disruption of TGF-β signaling (2) The model allows for to study of disruption in signaling pathways	(1) Requires surgical intervention and the use of magnets, which provides technical complexity (2) Unlike refractory human ulcers, this model heals over time (3) Inflammation can be partially caused by a foreign body (magnet)	[124,125]

**Table 3:** Recent studies on using BNPs to cure CWs.

BNPs	The Types of Cells	Production of NPs	Dosage Form	Degradation Period	Models	Results	Ref.
EV-AAV/MSC-Exo	HEK293T/hUC-MSCs	Density gradient ultracentrifugation-based on iodixanol (EV-AAV), Ultracentrifugation (MSC-Exo)	Thermosensitive hydrogel, FHCCgel	Up to 10–14 days	Analysis of 2D in vitro models: 1. Anti-inflammatory model (H_2_O_2_-treated HUVECs); 2. Cell scratch assay (HUVECs)	1. ↓ 8-OHDG level (*p* < 0.001), ↑ mPTP level (*p* < 0.001); 2. ↑ proliferative and migratory effects (*p* < 0.001)	[15]
					In vivo: Diabetic wound model (type 2 diabetic mice and male C57 mice)	↑ VEGF level (*p* < 0.001), ↓ wound healing time (*p* < 0.001), and ↑ collagen index (*p* < 0.001)	
DFATs-Exos	DFATs	Ultracentrifugation	Hydrogel based on gelatin methacrylate, GelMA	Within 14 days	Analysis on 2D in vitro models: 1. Tube formation assay (HUVECs) 2. Proliferation and migration assays in a high-glucose environment (HDFs)	1. ↑ tube formation (*p* < 0.01), ↑ VEGF and HGF levels (*p* < 0.001); 2. ↑ proliferation (*p* < 0.001), ↑ migration (*p* < 0.0001)	[24]
					In vivo: Diabetic wound model (BALB/c mice)	↑ wound closure rate (no statistics available), ↑ angiogenesis (evaluated CD31, *p* < 0.0001), ↑ proliferation (*p* < 0.05)	
ADSC-Exos	ADSCs	Differential ultracentrifugation	Injectable form for subcutaneous administration	No data	Analysis on 2D in vitro models: 1. Proliferation and migration assays in a high-glucose environment (HG RSF)	↑ proliferation (*p* < 0.05), ↑ migration (*p* < 0.05)	[7]
					In vivo: Diabetic wound model (SD rats)	↑ wound closure rate (*p* < 0.05) after 14 days, ↓ scar widths (*p* < 0.001), ↑ surface area and thickness of epithelial coverage (*p* < 0.001)	
MSCs-derived Exos	ADMSCs	Differential ultracentrifugation	Chitosan-PEG Hydrogel	Within 14 days	Analysis on 2D in vitro models: Only Exos: 1. Scratch wound assay (NIH/3T3) 2. Tube formation assay (HUVECs) CS-PEG-Exos: Proliferation assay using MTT (NIH/3T3)	1. ↑migration (*p* < 0.05), ↓ ROS level (*p* < 0.05) 2. ↑ loop formation (*p* < 0.05), tube length (*p* < 0.01), tube width (*p* < 0.001) ↑ proliferation (3 day: *p* <0.05, 5 day: *p* < 0.01)	[46]
FB-Exos	Dermal fibroblasts	Differential ultracentrifugation	Injectable form for subcutaneous administration	No data	Analysis on 2D in vitro models: 1. Tube formation assay (vECs) 2. Proliferation assay (vECs) 3. Apoptosis assay (vECs) 4. Scratch wound assay (vECs) 5. Transwell assays (vECs)	1. ↑tubulogenesis (*p* < 0.05), ↑ tubule formation (*p* < 0.001) 2. ↑ proliferation (immunofluorescence: *p* < 0.05, flow cytometry: *p* < 0.001) 3. ↓ apoptosis (immunofluorescence: *p* <0.001, flow cytometry: *p* < 0.001) 4. ↑ migration (*p* < 0.001) 5. ↑ migration (*p* < 0.01)	[31]
					In vivo: 1. Full-thickness excision wound model with GW4869 (C57BL/6 mice) 2. Diabetic wound model (C57BL/6 mice)	1. ↑ wound healing rate (*p* < 0.01), ↑ neovascularization (*p* < 0.001), and ↑ perfusion (*p* < 0.01) compared to GW4869-treated cells 2. ↑ wound healing rate (*p* < 0.001), ↑ neovascularization (*p* < 0.01), ↑ perfusion (*p* < 0.01)	
H-ADSCs-Exo	HypoxicADSCs	Differential ultracentrifugation	Injectable form for subcutaneous administration	No data	Analysis on 2D in vitro models: 1. CCK-8 assay (HUVEC and FR) 2. Transwell assays (HUVEC and FR) 3. Western blot analysis (HUVEC) 4. Tube formation assay (HUVEC)	H-ADSCs-Exo vs. PBS: 1.↑ proliferation (*p* < 0.001) 2.↑ migration (*p* < 0.001) 3. ↑ expression levels of VEGF, angiopoietin 1, collagen I, and fibronectin proteins (*p* < 0.001) 4.↑ length, number, and junction tubes (*p* < 0.001)	[50]
					In vivo: Diabetic wound model (SD rats)	H-ADSCs-Exo vs. PBS: ↓ ulcer size (*p* < 0.001), ↑ skin thickness (*p* < 0.001),↑ VEGF and CD31 (*p* < 0.001), ↓ TNF-α and IL-6 (*p* < 0.01)	
GelMA-EVs	HaCaT	Differential ultracentrifugation	Hydrogel based on gelatin methacrylate, GelMA	No data, but GelMA-EVs can be released approximately 14 days	Analysis on 2D in vitro models: 1. CCK-8 assay (HUVEC) 2. Transwell assays (HUVEC) 3. Wound healing assay (HUVEC) 4. Tube formation assay (HUVEC) 5. RT-qPCR (HUVEC)	GelMA-EVs vs. HaCaT-Evs: 1. ↑ proliferation (*p* < 0.05) 2. ↑ migration (*p* < 0.05) 3. ↑ wound closure rate (*p* < 0.05) 4. ↑ tube formation (*p* < 0.05) 5. ↑ mRNA expression levels of CD31 (*p* < 0.01), ANG-1(*p* < 0.05), and VEGF(*p* < 0.05)	[38]
					In vivo: Diabetic wound model (C57BL/6 mice)	GelMA-EVs vs. HaCaT-Evs: ↑ wound closure rate (*p* < 0.05), ↑ re-epithelialization (*p* < 0.05), and ↑ collagen deposition (*p* < 0.01)	
EMNVs transporting LncRNA-H19	HEK293 cells were transfected with an H19-OE lentiviral vector or an empty vector	Serial extrusion with two-step purification: ultrafiltration and density gradient ultracentrifugation based on a 30% sucrose/D2O cushion	Sodium Alginate Hydrogel, SAH	No data	Analysis on 2D in vitro models: 1. CCK-8 assay (HMEC-1) 2. Cell proliferation assay (HMEC-1) 3. Transwell assays (HMEC-1) 4. Tube formation assay (HMEC-1)	1. ↑ proliferation (*p* < 0.05) 2. ↑ proliferation (*p* < 0.05) 3. ↑ migration (*p* < 0.05) 4. ↑ percent of tube numbers and branch points (*p* < 0.05)	[40]
					In vivo: Diabetic wound model (SD rats)	↑ wound closure rate (*p* < 0.05),↑ length of renascent skin (*p* < 0.05), ↑ percentage of vessel area and vessel number (*p* < 0.05), and ↑ mature blood vessels (*p* < 0.05)	

**Table 4:** Clinical trials of BNPs for CWs.

Trial ID	Brief Title	Therapy	Delivery Method	Trial Phase	Results
NCT05475418	Pilot Study of Human Adipose Tissue-Derived Exosomes Promoting Wound Healing	Extracellular vesicles from adipose tissue	Sterile hydrogel + inert protective dressing	Not Applicable	No results posted
NCT04134676	Therapeutic Potential of Stem Cell Conditioned Medium on Chronic Ulcer Wounds	Wharton’s Jelly MSC Conditioned Medium (secretomes/exosomes)	Topical gel + transparent dressing	Phase 1	No results posted
NCT02565264	Effect of Plasma-Derived Exosomes on Cutaneous Wound Healing	Autologous plasma-derived exosomes	Direct application (exosome-rich buffer)	Early Phase 1	No results posted
NCT06812637	Efficacy and Safety of Wharton’s Jelly-Derived MSC Exosomes for Diabetic Foot Ulcers	WJ-MSC-derived exosomes	CMC-based gel	Phase 1	No results posted (Study completed 2024)
NCT06253975	Randomized Study of Extracellular Vesicles From Human Adipose Tissue Promoting Wound Healing	Adipose tissue-derived extracellular vesicles (AT-EVs)	HA hydrogel	Not Applicable	No results posted (Ongoing)
NCT06825884	Use of Extracellular Vesicles (EV) for Diabetic Foot Ulcers	Extracellular microvesicles (EV) from platelets	Perilesional injection (5 mL weekly) + exofiber dressing	Phase 1	No results posted (Ongoing)

## Data Availability

Not applicable.
